# Quantitative Methylation Level of the *EPHX1* Promoter in Peripheral Blood DNA Is Associated with Polycystic Ovary Syndrome

**DOI:** 10.1371/journal.pone.0088013

**Published:** 2014-02-05

**Authors:** Qing Sang, Xin Li, Haojue Wang, Huan Wang, Shaozhen Zhang, Ruizhi Feng, Yao Xu, Qiaoli Li, Xinzhi Zhao, Qinghe Xing, Li Jin, Lin He, Lei Wang

**Affiliations:** 1 State Key Laboratory of Genetic Engineering and MOE Key Laboratory of Contemporary Anthropology, School of Life Sciences, Fudan University, Shanghai, China; 2 Institutes of Biomedical Science, Fudan University, Shanghai, China; 3 Bio-X Center, Key Laboratory for the Genetics of Developmental and Neuropsychiatric Disorders, Ministry of Education, Shanghai Jiao Tong University, Shanghai, China; 4 Shanghai Ninth Hospital, Shanghai, China; 5 Department of Gynecology, Obstetrics and Gynecology Hospital of Fudan University, Shanghai, China; Institut national de la santé et de la recherche médicale, France

## Abstract

Steroid synthesis and metabolic pathways play important roles in the pathophysiology of PCOS, but until now there have been no studies on the methylation profiles of specific genes in steroid synthesis pathways that are known to be associated with PCOS. Here we used MassARRAY quantitative methylation analysis to determine the methylation levels of each CpG site or cluster in the promoters of *EPHX1*, *SRD5A1*, and *CYP11A1* in 64 peripheral blood samples. We further examined the methylation level of *EPHX1* in an independent cohort consisting of 116 people. Finally, we investigated the role of *EPHX1* in steroidogenesis in the KGN cell line. For *SRD5A1* and *CYP11A1*, there was no significant difference in methylation level between patients and controls. For *EPHX1*, however, the methylation levels of a few consecutive CpG sites and clusters were found to be significantly associated with PCOS. The methylation levels of a number of CpG clusters or sites were significantly lower in patients than in controls in the first cohort consisting of 64 people, such as clusters 13–14 (*P*<0.05), 15–16 (*P*<0.001), and 19–24 (*P*<0.001) and sites CpG_53 (*P*<0.01) and CpG_54 (*P*<0.05). Among differentiated methylation sites and clusters, the methylation levels of the CpG cluster 13–14 and CpG cluster 19–24 in PCOS patients were significantly lower than in controls in the second cohort of 116 people (*P*<0.05 for both). In addition, knockdown and overexpression experiments in KGN cells showed that *EPHX1* can regulate estradiol concentrations, and this indicates a role for *EPHX1* in steroidogenesis. Our study has demonstrated that methylation of the *EPHX1* promoter might be associated with PCOS. This study provides direct evidence that methylation plays an important role in PCOS and demonstrates a novel role for *EPHX1* in female reproduction.

## Introduction

Polycystic ovary syndrome (PCOS) is the most common endocrine disorder among reproductive-age women and has an estimated global prevalence of approximately 5%–10%. It is a leading cause of female infertility and is associated with polycystic ovaries, hirsutism, obesity, and insulin resistance [1], [2]. The etiology of PCOS is complex and is not well understood. Some evidence, however, suggests that genetic and environmental factors contribute to the etiology of PCOS [3].

Epigenetic regulation of gene expression is important in human phenotype expression and has been implicated in various diseases [4]–[6]. However, the role of epigenetics in PCOS is only now being elucidated. Demethylation of the luteinizing hormone receptor (LHR) gene has been identified in a mouse model of PCOS [7], and skewed X-chromosome inactivation has been demonstrated in human PCOS patients [8], [9]. Xu et al. performed pioneering work on the global DNA methylation status of peripheral blood from PCOS patients and healthy women. Although no significance differences in DNA methylation were seen in the peripheral blood in their study, further study of specific genes in specific tissue was recommended [10]. We recently investigated the methylation level of *follistatin* in the peripheral blood of patients with PCOS but did not find any difference compared to the methylation level in controls [11].

*CYP11A1*, *EPHX1*, *HSD17B6*, *CYP17*, *CYP19a1*, and *SRD5A1* are key genes in steroid synthesis and metabolic pathways that play important roles in the pathophysiology of PCOS [12]–[14]. Studies have investigated whether polymorphisms in these key genes confer PCOS susceptibility [15]–[18]. Using a CpG island searcher (http://www.uscnorris.com/cpgislands2/cpg.aspx), we found that *SRD5A1*, *CYP11A1*, and *EPHX1* possess obvious CpG islands in their promoter regions.

In this study, we used MassARRAY techniques to determine whether there is an association between the methylation levels of the promoters of *SRD5A1*, *CYP11A1*, and *EPHX1* and the etiology of PCOS. In addition, we used knockdown and overexpression of *EPHX1* in cells to investigate the function of this gene in estradiol (E_2_) synthesis. Our results provide new evidence for the important role of epigenetics in the etiology of PCOS and provide new insights into the function of *EPHX1*.

## Materials and Methods

### 2.1 Patients and Peripheral Blood Collection

In the first cohort, 32 PCOS patients (mean age (SEM) = 26.8±0.2 years) and 32 healthy controls (mean age (SEM) = 27.2±0.1 years) were recruited from the outpatient clinic of the Xi’an Fourth Hospital. In the second cohort, 49 PCOS patients (mean age (SEM) = 28±0.2 years) and 67 controls (mean age (SEM) = 28±0.1 years) were recruited from the outpatient clinic of the Shanghai Ninth Hospital. The diagnosis of PCOS was based on the following revised Rotterdam diagnostic criteria [19]: (i) oligo-ovulation and/or anovulation; (ii) clinical and/or biochemical signs of hyperandrogenism; and (iii) polycystic ovaries. Women who met at least two of these criteria were defined as having PCOS. Oral contraceptives or other hormonal medications had not been taken by any of the patients within the previous 3 months, and blood samples were taken from all of the patients after a 12-hour fast. The Fudan University Ethics Review Committee approved the study, and written informed consent was obtained from all participants.

### 2.2 DNA Preparation and CT Conversion

Genomic DNA from the peripheral blood of PCOS patients and controls was isolated using the QIAamp DNA Mini Kit (Qiagen, Hilden, Germany). Bisulfite conversion of the genomic DNA was performed with the EZ DNA CT Conversion Reagent, Zymo Research Corporation (Irvine, CA, US) as recommended by the manufacturer.

### 2.3 MassARRAY Quantitative Methylation Analysis

MassARRAY is a novel EpiTYPER assay for high-throughput analysis of DNA methylation patterns using matrix-assisted laser desorption/ionization time-of-flight mass spectrometry (MALDI-TOF MS). This assay is a tool for the detection and quantitative analysis of DNA methylation using MALDI-TOF MS and the MassCLEAVE reagent, which enables base-specific (C/T) cleavage reactions [20]. PCRs were carried out in a volume of 5 µL containing 1 µL (10 ng) bisulfite-treated template DNA using PCR polymerase (Sequenom, Inc., San Diego, CA, USA). The protocol consisted of a 4 minute denaturing step at 95°C; 45 cycles of 95°C for 20 seconds, 56°C for 30 seconds, and 72°C for 1 minute. There was a final elongation step of 3 minutes at 72°C. For partially methylated CpG sites identified by sequencing analysis, quantitative methylation was further measured using the MassARRAY Compact System following the MassCLEAVE training protocol (Sequenom). This system uses bisulfite-converted genomic DNA and combines MassCLEAVE base-specific cleavage with MALDI-TOF mass spectrometry. The resulting methylation calls were analyzed with the EpiTYPER software package (Sequenom) to generate quantitative CpG methylation results (http://www.sequenom.com). All the primers used were listed in Table S1.

### 2.4 Cell Culture

Steroidogenic human granulosa-like KGN tumor cells were kindly donated by Dr. Fei Sun from the University of Science and Technology (Hefei, China) [21]. The cells were grown in HyClone DMEM high-glucose medium (HyClone Laboratories, Inc., Utah, USA) with 10% fetal bovine serum (Life Technologies, Inc., Carlsbad, CA) and 1% antibiotics (100 U/mL penicillin and 100 µg/mL streptomycin; HyClone Laboratories, Inc.) at 37°C under a 5% CO_2_ atmosphere. The culture medium was changed every other day.

### 2.5 Plasmid and siRNA

The pMCB3 overexpression vector was kindly donated by Dr. Shimin Zhao from Fudan University (Shanghai, China). Full-length human *EPHX1* cDNA was amplified from the cDNA of the KGN cells and subcloned into the pMCB3 vector through BamH1 and Xho1 restriction sites. All constructs were confirmed by DNA sequencing. Chemically synthesized *EPHX1* siRNA and scrambled or control siRNA were purchased from Shanghai GenePharma (Shanghai, China). The sequence of the siRNA is shown in Table S2. Annealed siRNA duplexes were resuspended in RNAse-free solution buffered to pH 7.4.

### 2.6 Transient Transfection and Hormone Analysis

KGN cells were plated in 24-well plates and grown to 70%–80% confluence. The culture medium was changed 3 h to 4 h before transfection. Transient transfection with plasmid DNA was performed with Lipofectamine 2000 (Invitrogen) following the manufacturer’s protocol. Transient transfection of siRNA was performed with HiPerFect (Qiagen Sciences, Germantown, MD). The culture medium was replaced with serum-free medium 24 h after transfection, and 10 nM testosterone (Biodee BioTech Corporation, Beijing, China) was added to each well for another 24 h. After this final incubation, the culture medium was collected and centrifuged at 1000×*g* for 5 min to prepare it for hormone analysis.

The remaining cells on the 24-well plate were lysed with Ripo (Biocolor BioScience & Technology Company, Shanghai, China) and centrifuged at 12,000×*g* for 10 minutes at 4°C and the supernatant was collected. Total protein concentration was measured with a BCA-100 Protein Quantitative Analysis Kit (Biocolor BioScience & Technology Company) and an Infinite M200 PRO reader (Tecan, Switzerland).

Concentrations of E_2_ and progesterone (P) in the culture medium were measured with the UniCel® DxI 800 Immunoassay System (Beckman Coulter, Inc., Brea, CA), which is an automated random-access chemilluminescence-based assay. The intra-assay and inter-assay coefficients of variation were less than 10% and 15%, respectively. The E_2_ concentration was further normalized to the total protein concentration in each well.

### 2.7 Statistical Analysis

The percent methylation of each CpG site or CpG cluster was expressed as the mean ± SEM. The data were analyzed using Student’s t-test with statistical significance at the level of *P*<0.05. Multiple testing was controlled by Bonferroni correction.

## Results

### 3.1 Clinical Parameter Evaluation

The clinical characteristics of the PCOS patients and healthy controls are shown in Table 1 and Table 2, respectively. The age, height, weight, and serum concentrations of E_2_, P, and prolactin were comparable between the two groups, but serum luteinizing hormone, follicle stimulating hormone, and testosterone concentrations were significantly different. The PCOS group had much higher serum levels of luteinizing hormone and testosterone than the control group, and the serum level of follicle stimulating hormone was much lower in the PCOS group than control group.

**Table 1:** Clinical characteristics of healthy controls and PCOS patients in the first cohort (Control n = 32, PCOS n = 32).

Variable	Control	PCOS
**Age (years)**	26.8±0.2	27.2±0.1
**BMI (kg/m^2^)**	20.6±0.3	21.3±0.5
**LH (mlU/mL)**	2.8±0.3	6.2±1.2^a^
**FSH (mlU/mL)**	7.3±0.6	5.9±0.3^a^
**E_2_ (pg/mL)**	41.7±6.1	51.6±7.7^a^
**PRL (ng/mL)**	23.0±1.5	20.5±1.4
**P (ng/mL)**	1.0±0.1	1.1±0.1
**T** **(ng/mL)**	0.3±0.0	0.6±0.1^a^

**Table 2:** Clinical characteristics of healthy controls and PCOS patients in the second cohort (Control n = 49, PCOS n = 67).

Variables	Control	PCOS
**Age (year)**	28.0±0.2	28.0±0.1
**BMI (kg/m^2^)**	20.8±0.2	22.2±0.3^a^
**LH (mIU/mL)**	3.1±0.1	5.3±0.5^a^
**FSH (mIU/mL)**	6.9±0.3	5.9±0.2^a^
**E_2_ (pg/mL)**	44.4±4.7	48.0±3.6^a^
**PRL (ng/mL)**	26.2±1.6	26.0±2.1
**P (ng/mL)**	1.2±0.2	1.5±0.4
**T (ng/mL)**	0.3±0.0	0.4±0.0^a^

Data represent the mean ± SEM;

BMI: body mass index, LH: luteinizing hormone, FSH: follicle stimulating hormone, E_2_: estradiol, PRL: prolactin, P: progesterone, T: testosterone;

a *p*<0.001 compared with the control group, *p* values are determined by Student’s *t*-test.

### 3.2 MassARRAY Quantitative Methylation Analysis of the *EPHX1*, *SRD5A1*, and *CYP11A1* Promoters

**Figure 1:**
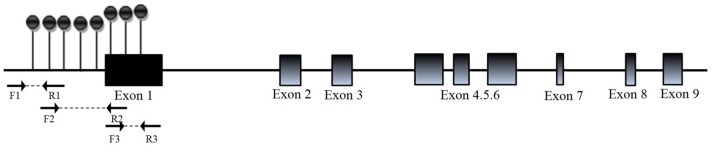
The schematic diagram of the CpG sites in the *EPHX1* promoter. CpG sites are depicted by lollipop markers. Binding sites for the forward and reverse primers are shown as arrows below the diagram. The number of lollipop markers is not indicative of the number of detected CpG sites, and 47 informative CpG sites were identified in the *EPHX1* promoter region.

**Figure 2:**
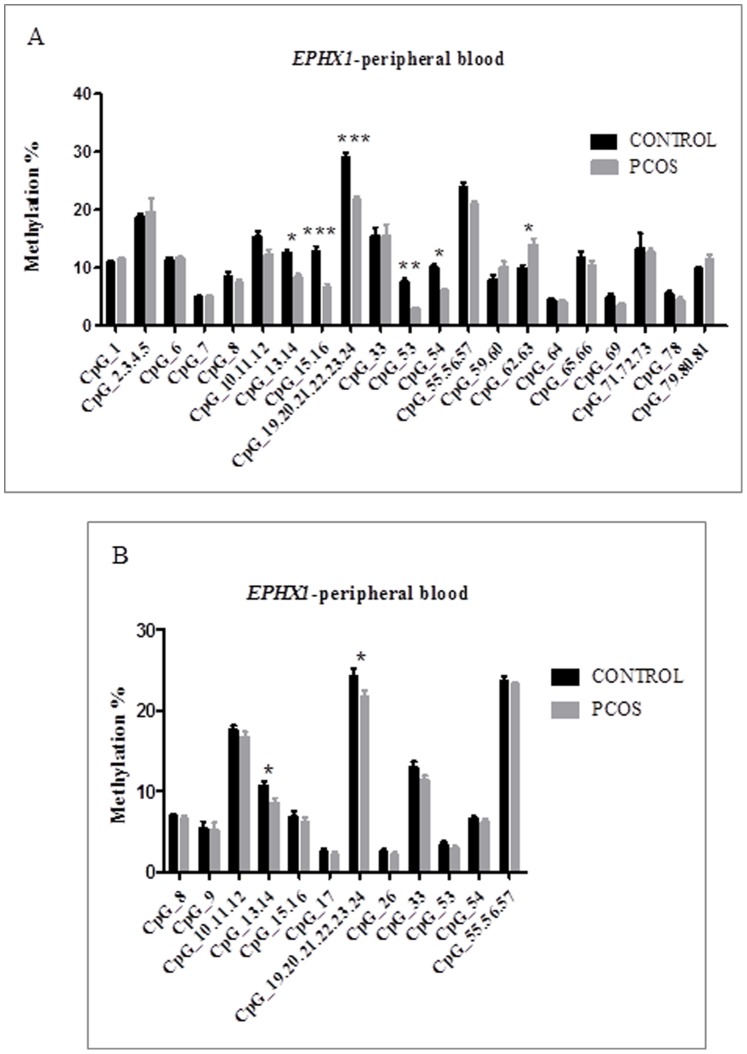
The mean methylation levels for each CpG site of the *EPHX1* promoter in PCOS patients and healthy women in the first cohort consisting of 64 peripheral blood samples (A) and in the second cohort consisting of 116 peripheral blood samples (B). Values are mean ± SEM. *** represents *p*<0.001, ** represents *p*<0.01, * represents *p*<0.05.

We first investigated the methylation level of each CpG site or cluster in the promoters of *EPHX1*, *SRD5A1*, and *CYP11A1* in 64 peripheral blood DNA samples (32 PCOS patients and 32 healthy controls). For SRD5A1 and CYP11A1, there were no significant difference in methylation of any promoter CpG site or cluster between PCOS patients and healthy women (Figure S1). The promoter of *EPHX1* contained 81 CpG sites (Figure S3), and the EpiTYPER software was used to design three pairs of primers as shown in Figure 1 and Table S1. There were significant differences in methylation levels of some CpG sites or clusters in the *EPHX1* promoter between PCOS patients and controls. As shown in Figure 2, the average methylation level of the CpG clusters 13–14, 15–16, and 19–24 was 18% in controls and 12% in PCOS patients, and the average methylation level of the CpG sites 53 and 54 and CpG cluster 55–57 was 14% in controls and 12% in PCOS patients (*P*<0.05) (Table 3 and Figure 2A).

**Table 3:** The methylation status (methylation %) of the CpG sites in the promoter region of *EPHX1* between healthy women and PCOS patients in peripheral blood samples from the first cohort (Control n = 32, PCOS n = 32).

CpG sites	Control	PCOS	*Delta value*	*p*-value
***EPHX1*** **_CpG_1**	10.8±0.4	11.4±0.3	0.6	0.30
***EPHX1*** **_CpG_2.3.4.5**	18.7±0.5	19.6±2.4	0.9	0.71
***EPHX1*** **_CpG_6**	11.3±0.5	11.7±0.3	0.4	0.52
***EPHX1*** **_CpG_8**	4.9±0.2	5.0±0.3	0.1	0.63
***EPHX1*** **_CpG_9**	8.5±0.9	7.5±0.4	1.0	0.29
***EPHX1*** **_CpG_10.11.12**	15.3±0.9	12.3±0.8	3.0	0.12
***EPHX1*** **_CpG_13.14**	12.5±0.5	8.4±0.5	4.1	<0.05
***EPHX1*** **_CpG_15.16**	12.8±0.8	6.6±0.5	6.2	<0.001
***EPHX1*** **_CpG_19-24**	28.9±0.9	21.6±0.6	7.3	<0.001
***EPHX1*** **_CpG_25**	0.7±0.4	0.7±0.2	0.0	0.85
***EPHX1*** **_CpG_27**	2.2±0.6	2.1±0.4	0.1	0.80
***EPHX1*** **_CpG_34**	14.9±1.5	18.0±3.2	3.1	0.38
***EPHX1*** **_CpG_53**	7.3±0.6	2.8±0.3	4.5	<0.01
***EPHX1*** **_CpG_54**	10±0.6	6.0±0.3	4.0	<0.05
***EPHX1*** **_CpG_55.56.57**	24±0.7	21.0±0.4	3.0	0.16
***EPHX1*** **_CpG_59.60**	8±0.8	10.0±1.0	2.0	0.13
***EPHX1*** **_CpG_61**	4.7±0.3	4.9±0.6	0.2	0.73
***EPHX1*** **_CpG_62.63**	9.9±0.5	13.8±1.2	3.9	<0.05
***EPHX1*** **_CpG_64**	4.5±0.3	4.1±0.4	0.4	0.39
***EPHX1*** **_CpG_65.66**	11.7±0.9	10.4±0.7	1.3	0.27
***EPHX1*** **_CpG_67.68**	2.6±0.2	2.8±0.4	0.2	0.71
***EPHX1*** **_CpG_69**	4.9±0.5	3.5±0.3	1.4	0.19
***EPHX1*** **_CpG_71.72.73**	13.3±2.6	12.7±0.6	0.6	0.81
***EPHX1*** **_CpG_78**	5.4±0.6	4.2±0.6	1.2	0.15
***EPHX1*** **_CpG_79.80.81**	9.7±0.5	11.5±0.7	1.8	0.22

Data represent the mean ± SEM, *p* values are determined by two-way ANOVA with Bonferroni post-tests.

To confirm this difference, we further validated the significance of the results in a second cohort consisting of 67 PCOS patients and 49 controls. We found that there were still two CpG clusters (CpG cluster 13–14 and the CpG cluster 19–24) that were significantly different between PCOS patients and controls. The average methylation levels of CpG cluster 13–14 were 10.7% in controls and 8.5% in PCOS patients (*P*<0.05), and the average methylation levels of CpG cluster 19–24 were 24.3% in controls and 21.5% in the PCOS patients (*P*<0.05) (Table 4 and Figure 2B).

**Table 4:** The methylation status (methylation %) of the promoter region covered by the second *EPHX1* primer set in the peripheral blood samples from healthy women and PCOS patients in the second cohort (Control = 49, PCOS = 67).

CpG sites	Control	PCOS	*Delta value*	*p*-value
***EPHX1*** **_CpG_8**	6.9±0.3	6.7±0.4	0.2	0.72
***EPHX1*** **_CpG_9**	5.2±0.8	5.5±0.7	0.3	0.89
***EPHX1*** **_CpG_10.11.12**	17.6±0.5	16.8±0.6	0.8	0.31
***EPHX1*** **_CpG_13.14**	10.7±0.5	8.5±0.5	2.2	<0.05
***EPHX1*** **_CpG_15.16**	6.9±0.7	6.5±0.5	0.4	0.71
***EPHX1*** **_CpG_17**	2.4±0.4	2.1±0.3	0.3	0.44
***EPHX1*** **_CpG_19-24**	24.3±0.9	21.4±0.8	2.9	<0.05
***EPHX1*** **_CpG_26**	2.4±0.4	2.1±0.3	0.3	0.44
***EPHX1*** **_CpG_33**	12.9±0.7	11.6±0.5	1.3	0.12
***EPHX1*** **_CpG_53**	3.4±0.3	3.2±0.3	0.2	0.64
***EPHX1*** **_CpG_54**	6.6±0.3	6.2±0.3	0.4	0.27
***EPHX1*** **_CpG_55.56.57**	23.7±0.5	23±0.3	0.7	0.22

Data represent the mean ± SEM, *p* values are determined by two-way ANOVA with Bonferroni post-tests.

### 3.3 Role of *EPHX1* in E_2_ Synthesis in KGN Cells

**Figure 3:**
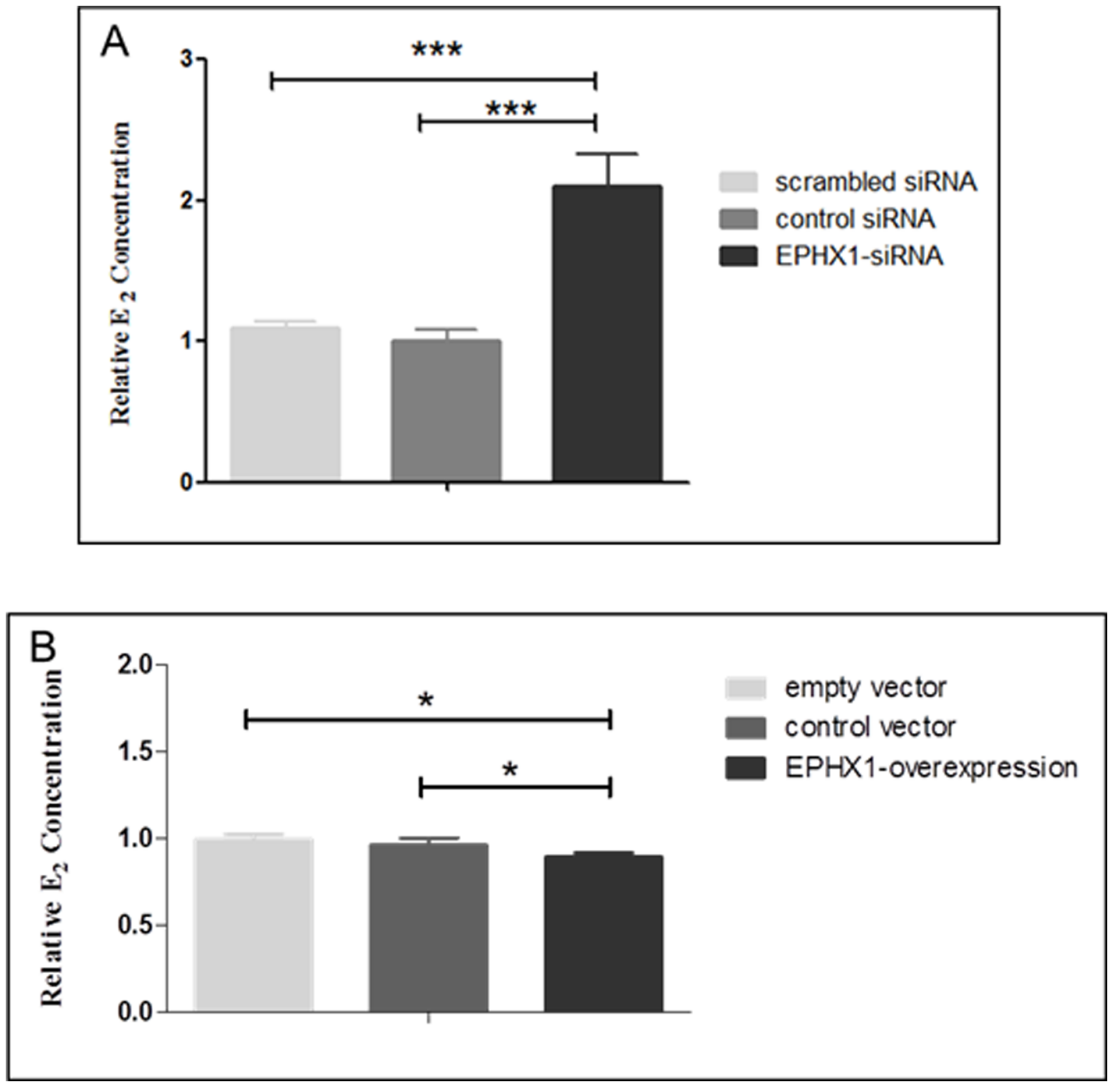
*EPHX1* affects E_2_ synthesis in the KGN cell line. The E_2_ level is increased when cells are transfected with *EPHX1* siRNA (A). The E_2_ level is decreased when cells are transfected with the *EPHX1* cDNA plasmid (B). *** represents *p*<0.001 and * represents *p*<0.05.

To investigate the function of *EPHX1,* KGN cells were transfected with either siRNA against *EPHX1* or with a plasmid carrying the cDNA for *EPHX1*. As shown in Figure S2, the *EPHX1* mRNA expression level was decreased when cells were transfected with the siRNA, and its mRNA expression level was elevated when the cells were transfected with the *EPHX1* cDNA plasmid. Compared to cells transfected with negative control or scrambled siRNA, cells transfected with siRNA against *EPHX1* had significantly higher E_2_ concentrations. On the contrary, when KGN cells were transfected with the *EPHX1* cDNA plasmid, the E_2_ level was significantly lower than in cells transfected with control vector or empty vector (**Figure 3**).

## Discussion

Epigenetic changes have been found to be associated with common diseases such as type 2 diabetes, various kinds of cancers, and mental disorders such as schizophrenia and depression. Recent advances in epigenetics and epigenomics have provided evidence that epigenetic mechanisms may function as an interface between environmental factors and the genome [22].

PCOS is a multigenic disorder, and both genetic and environmental factors play a role in its etiology and pathophysiology. Xu et al conducted a pilot epigenetic study of DNA methylation in PCOS patients by comparing the global methylation levels between these patients and age-matched healthy controls [10]. Although there was no significant difference in the global methylation of peripheral leukocyte DNA between PCOS patients and controls, the authors of that study suggested that the methylation levels in specific genes and key tissues were worthy of further investigation. More recently, they have performed a genome-wide methylation analysis and identified the first specific alterations in the epigenome of infant and adult rhesus monkeys that have been exposed to androgenization. These results support the potential contributory role of epigenomic perturbations in the etiology of PCOS [23].

In the current study, we focused on three critical genes in steroid synthesis and metabolic pathways and systematically analyzed the methylation status of their promoters in the peripheral blood of PCOS patients and healthy controls. We found that the methylation level of a subset of CpG clusters in the promoter region of the *EPHX1* gene was lower in PCOS patients than in controls. We also found that *EPHX1* regulated E_2_ levels in KGN cells. Our study is the first to show that *EPHX1* plays an important role in steroidogenesis in vitro and that the methylation status of its promoter has a close relationship with PCOS.

*EPHX1* encodes epoxide hydrolase 1, which is a critical biotransformation enzyme that converts epoxides from the degradation of aromatic compounds into trans-dihydrodiols that can be conjugated and excreted from the body [24]. This protein plays a key role in detoxification processes and in the metabolism of endogenous and exogenous compounds. Several studies have shown that *EPHX1* has an important effect on the female reproduction system and influences susceptibility to spontaneous abortion [25], ovarian cancer [26], and preeclampsia [27]. Furthermore, Korhonen et al demonstrated that two exonic single nucleotide polymorphisms of *EPHX1* are associated with PCOS [28] suggesting that *EPHX1* has a role in the etiology of PCOS.

This is direct evidence that EPHX1 can regulate E_2_ concentrations in vitro. The ability of granulosa cells to convert testosterone to E_2_ is an important indicator for PCOS because this process contributes to hyperandrogenism, which is the most important clinical symptom for PCOS diagnosis [29]–[32]. Taken together, our results suggest that the reduced methylation level of the *EPHX1* promoter region in PCOS patients might activate *EPHX1* expression, which in turn could suppress E_2_ production from testosterone and increase the risk of PCOS. Contrary to our in vitro findings, the in vivo E_2_ level is higher in PCOS patients than in the control. We speculate, therefore, that the in vivo E_2_ level might not be determined simply by *EPHX1* gene expression. Instead, it might be affected by multiple factors, such as higher testosterone level or abnormalities in other metabolic traits.

Because RNA from peripheral blood was not obtained, we could not directly evaluate the *EPHX1* expression level and this is a limitation of the current study. There is also evidence that methylation profiles might be different in different cell types and tissues. In this study, methylation of peripheral blood DNA was investigated. Although peripheral blood is not the target tissue for PCOS, it might provide useful clues for elucidating the disease mechanism and the changes in methylation that we have identified might also stimulate future research. Methylation levels in peripheral blood DNA have been widely investigated in various diseases, including diabetes [33], obesity [34], major depression [35], and various cancers [36]–[38]. This evidence for the role of methylation in other diseases strengthened the rationale for studying methylation in peripheral blood DNA in relation to PCOS.

Glossop et al demonstrated that DNA methylation pattern vary dramatically between blood cell types [39]. In our study, we cannot distinguish DNA from specific cell types in the whole blood. Thus, we cannot exclude the possibility that the differentiated methylation level might reflect differences in different cell types. However, the differentiated methylation level of the promoter region of the gene was validated by the two independent cohorts, and this increases the possibility that differentiated methylation levels in regions of the gene might reflect the difference between controls and PCOS patients.

Although differences observed in the study at significant loci in the larger cohort are low (<3%), the significant loci associated with PCOS are in the proximity of the transcription start site (TSS), which might have an important impact on corresponding gene expression. For example, several transcriptional factors such as GAGA-3, Adf-1, and StuAp might bind to differentially methylated CpG cluster 19–24. The change in methylation level of the CpG clusters might affect binding of those transcription factors, which in turn might have roles in the development and progression of PCOS. This, along with our results showing that E_2_ levels were affected by *EPHX1* expression, provides convincing evidence that the methylation level of *EPHX1* is associated with PCOS.

In summary, we have found significant differences in the methylation level of the *EPHX1* promoter region in peripheral blood between PCOS patients and healthy controls. We have also shown that *EPHX1* can regulate estradiol levels in granulosa cells. This is the first study to provide direct evidence that the methylation level of *EPHX1* is associated with PCOS. More in-depth studies of the molecular mechanisms of methylation of *EPHX1* in PCOS, and analysis of the methylation levels of other related genes, might yield new insights into the pathophysiology of this disorder.

## Supporting Information

Figure S1 **Comparison of mean methylation levels for each CpG site in the** ***CYP11A1*** **(A) and** ***SRD5A1*** **(B) promoters between PCOS patients and healthy women.** Values are the mean ± SEM.
(TIF)

Figure S2 **Relative mRNA expression of the** ***EPHX1*** **gene in KGN cells transfected with** ***EPHX1*** **siRNA and** ***EPHX1*** **cDNA plasmid.** The final data were normalized to human GAPDH. ** represents *p*<0.01 and * represents *p*<0.05.
(TIF)

Figure S3 **Positions of the CpGs in the individual CpG clusters that we detected by MassARRAY.** CpG clusters are labeled with yellow or green. Base C in CpGs are labeled with red.
(TIF)

Table S1 **The primer sets of methylation analysis used in this study.**
(DOCX)

Table S2 **Sequence of** ***EPHX1*** **-targeted siRNA.**
(DOCX)
