# Supplementary figures and images for: Quantitative Methylation Level of the EPHX1 Promoter in Peripheral Blood DNA Is Associated with Polycystic Ovary Syndrome
Source: PLoS One. 2014 Feb 5;9(2):e88013. doi: 10.1371/journal.pone.0088013 (PMC3914883; doi:10.1371/journal.pone.0088013)

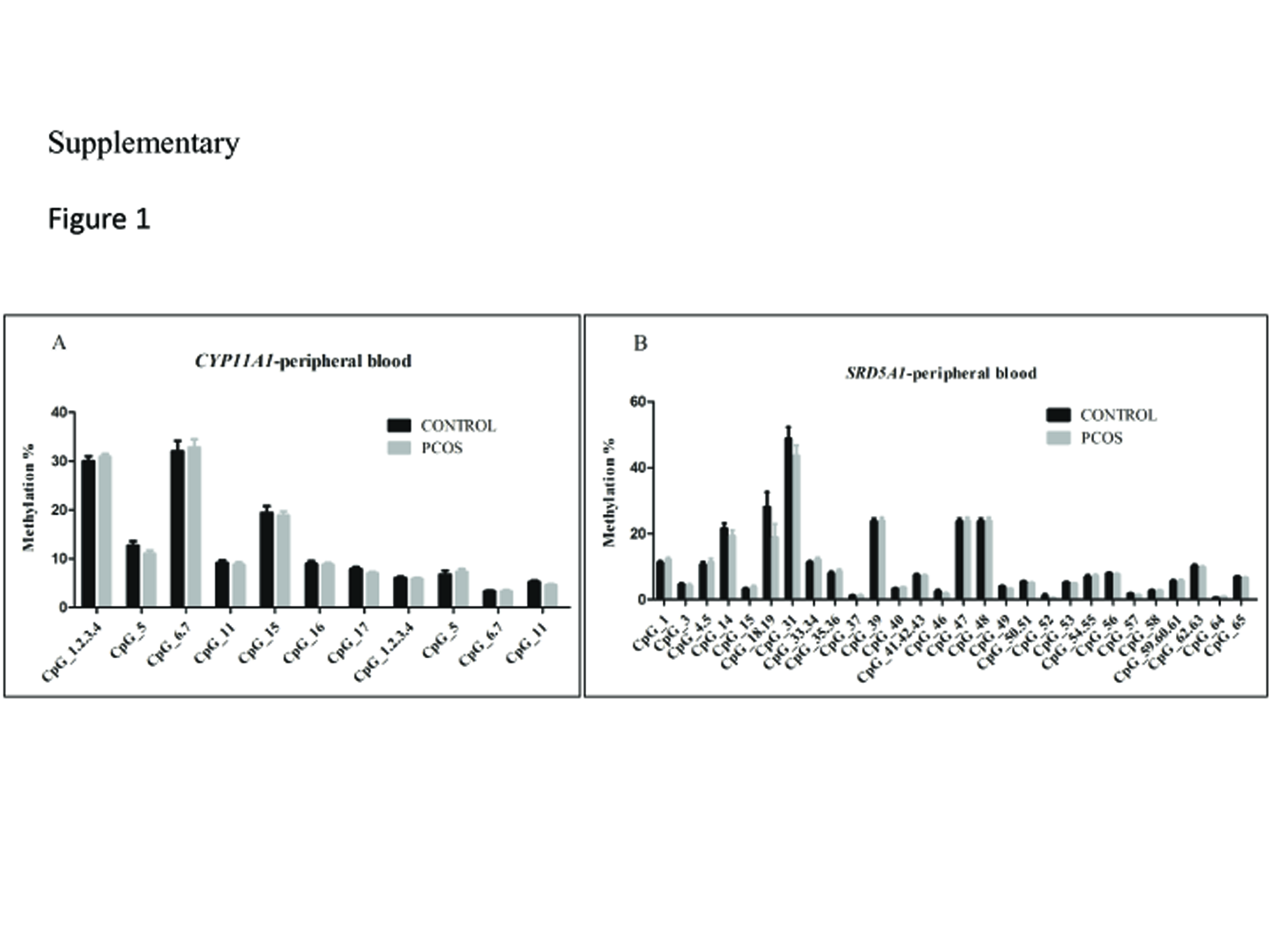

Supplement: Figure S1 — Comparison of mean methylation levels for each CpG site in the CYP11A1 (A) and SRD5A1 (B) promoters between PCOS patients and healthy women. Values are the mean ± SEM. (TIF) [file pone.0088013.s001.tif]

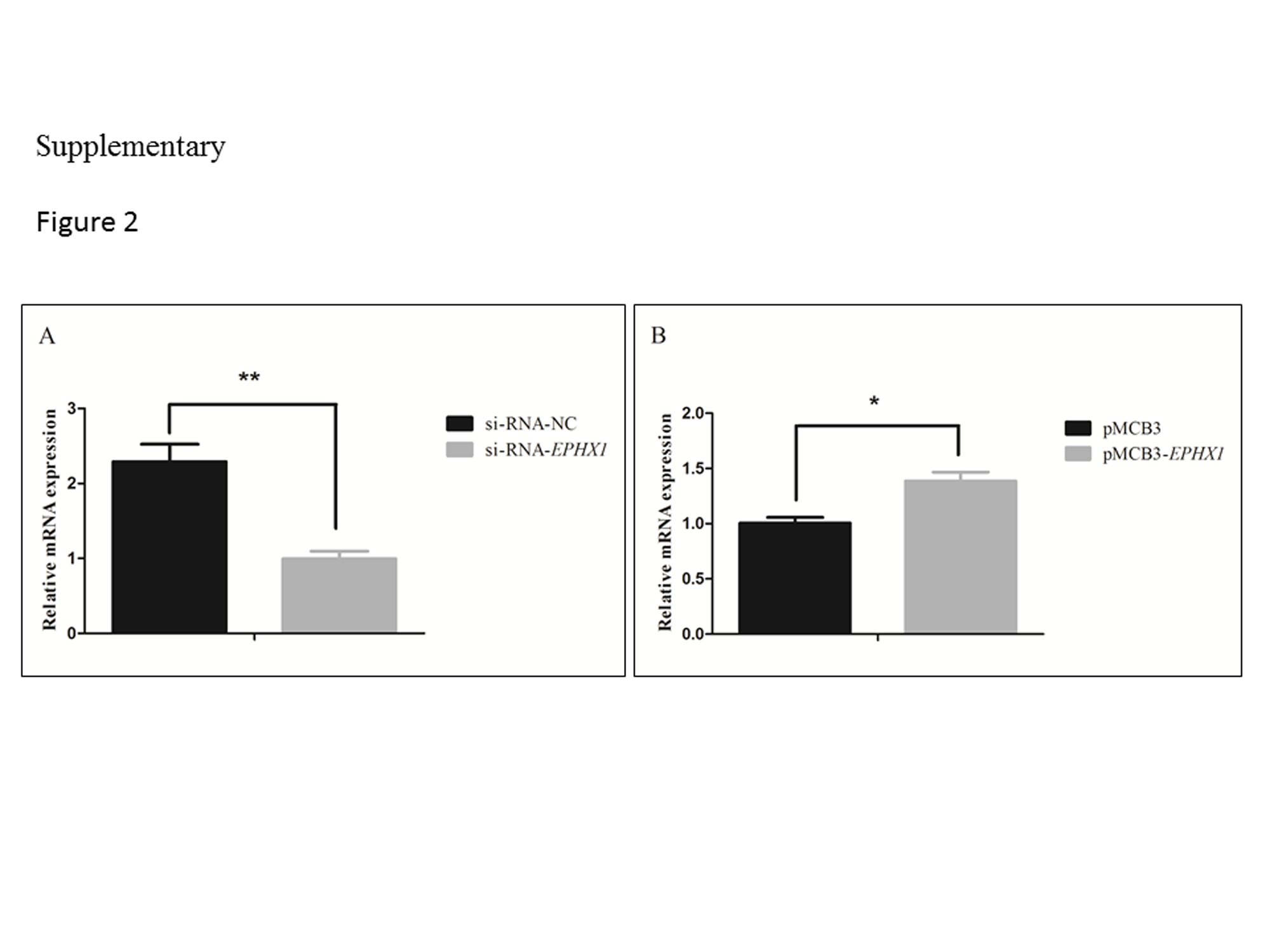

Supplement: Figure S2 — Relative mRNA expression of the EPHX1 gene in KGN cells transfected with EPHX1 siRNA and EPHX1 cDNA plasmid. The final data were normalized to human GAPDH. ** represents p<0.01 and * represents p<0.05. (TIF) [file pone.0088013.s002.tif]

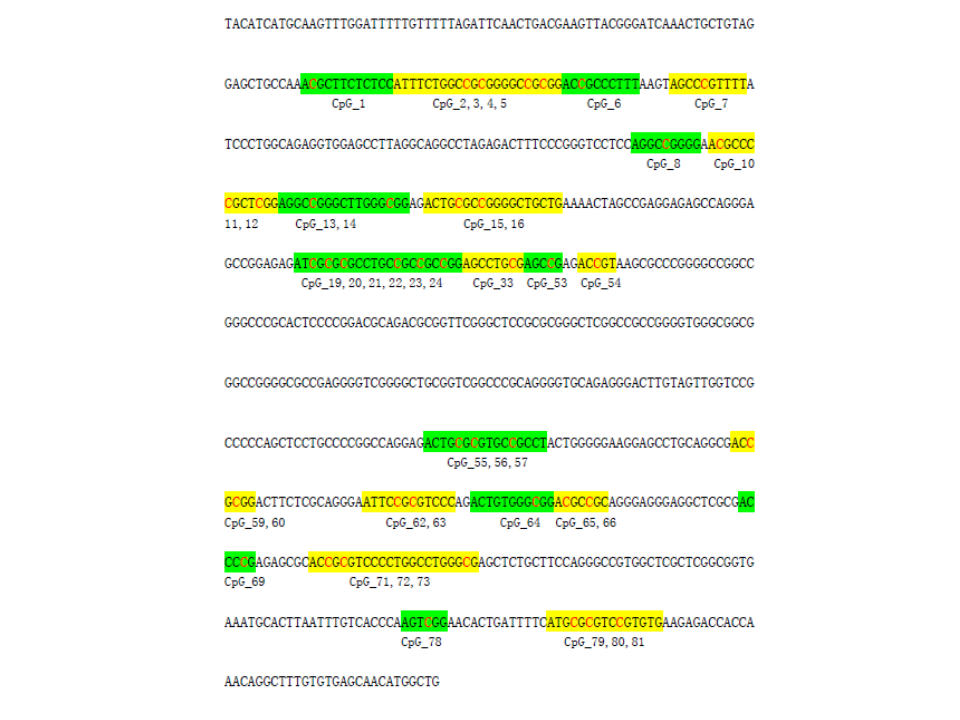

Supplement: Figure S3 — Positions of the CpGs in the individual CpG clusters that we detected by MassARRAY. CpG clusters are labeled with yellow or green. Base C in CpGs are labeled with red. (TIF) [file pone.0088013.s003.tif]
