# Supplementary material for: Quantitative Methylation Level of the EPHX1 Promoter in Peripheral Blood DNA Is Associated with Polycystic Ovary Syndrome
Source: PLoS One. 2014 Feb 5;9(2):e88013. doi: 10.1371/journal.pone.0088013 (PMC3914883; doi:10.1371/journal.pone.0088013)
Supplement: Table S1 — The primer sets of methylation analysis used in this study. (DOCX) [file pone.0088013.s004.docx]

**Table S1**. The primer sets used in this study

| **Name** | **Sequence (5'→3')** |
| --- | --- |
| ***EPHX1*-01-F** | aggaagagagGGGATTAAATTGTTGTAGGAGTTG |
| ***EPHX1*-01-R** | cagtaatacgactcactatagggagaaggctTAAACCTACCTAAAACTCCACCTCT |
| ***EPHX1*-02-F** | aggaagagagGGTAGAGGTGGAGTTTTAGGTAGGT |
| ***EPHX1*-02-R** | cagtaatacgactcactatagggagaaggctCTACAAACTCCTTCCCCCAATAAAC |
| ***EPHX1*-03-F** | aggaagagagGTTTATTGGGGGAAGGAGTTTGTAG |
| ***EPHX1*-03-R** | cagtaatacgactcactatagggagaaggctCAACCATATTACTCACACAAAACCTA |
