# Supplementary material for: Quantitative Methylation Level of the EPHX1 Promoter in Peripheral Blood DNA Is Associated with Polycystic Ovary Syndrome
Source: PLoS One. 2014 Feb 5;9(2):e88013. doi: 10.1371/journal.pone.0088013 (PMC3914883; doi:10.1371/journal.pone.0088013)
Supplement: Table S2 — Sequence of EPHX1 -targeted siRNA. (DOCX) [file pone.0088013.s005.docx]

**Table S2**. Sequence of *EPHX1*-targeted siRNA.

|  | **Sequence** |
| --- | --- |
| **Sense** | CCAAAGCUCAUCUCCUAUUTT |
| **Antisense** | AAUAGGAGAUGAGCUUUGGTT |
